# Co-application of biochar and phosphorus increases soil microbial biomass, mycorrhizal colonization, growth, and nutrition of subterranean clover

**DOI:** 10.3934/microbiol.2025004

**Published:** 2025-01-10

**Authors:** Zakaria M. Solaiman, Paul Blackwell, Muhammad Izhar Shafi, Nariman D. Salman, Paul Storer, Emre Babur

**Affiliations:** 1 UWA School of Agriculture and Environment, and The UWA Institute of Agriculture, The University of Western Australia, Perth, WA 6009, Australia; 2 Retired from the Department of Primary Industries and Regional Development, PO Box 110, Geraldton, WA 6530, Australia; 3 Department of Soil and Environmental Sciences, The University of Agriculture, Peshawar, 25000, Pakistan; 4 Department of Soil Sciences and Water Resources, College of Agriculture, Baghdad University, Iraq; 5 Troforte Innovations Pty Ltd, Wangara, WA 6065, Australia; 6 Soil and Ecology Department, Faculty of Forestry, Kahramanmaras Sutcu Imam University, Kahramanmaras 46050, Turkey

**Keywords:** Biochar, phosphorus, soil properties, subterranean clover, nodulation, mycorrhizal fungi

## Abstract

Phosphorus (P) plays important roles in the arbuscular mycorrhizal (AM) colonization and rhizobium nodulation processes. Additionally, biochar's positive roles in mycorrhizal colonization and nodulation are articulated. However, the effect of the co-application of biochar and P on AM colonization and rhizobium nodulation was poorly studied. This study investigated the effect of the co-application of wheat straw biochar and P using soil columns on mycorrhizal colonization, nodulation, and the growth of subterranean clover. The soil was amended with wheat straw biochar at 0, 5, and 10 t ha^−1^ with different levels of P fertilizer at 0, 5, and 10 kg P ha^−1^. These studies showed that adding biochar at 5 t ha^−1^ along with mineral P fertilizer increased plant growth, mycorrhizal root colonization and nodulation, and P concentration in plants. In most cases, the increasing trend of the biomass yield was higher when biochar and the P fertilizer were applied together at a higher level (P10). These findings suggested that an increased biochar application rate can increase the subterranean clover growth in soil with either no (P0) or a lower P (P5) fertilizer application. Mycorrhizal colonization could help to improve the P supply and subsequently stimulate the root nodulation of leguminous plants.

## 1. Introduction

Intensive agricultural practices such as overgrazing, deforestation, the increasing world population, limited freshwater, land resources, mining, and manufacturing industries have created challenging dialogues to discuss the sustainable strengthening of agriculture [1],[2]. Despite the agricultural productivity growth rapidly expanding over the last 50 years, the long-term projection rate of the crop yield growth for the most important crops, resources, and soil nutrients has declined. The low availability of essential plant nutrients and the subsequent decline in yield can adversely impact food security and economic development. Therefore, to enhance the soil nutrient supply and the crop yield, the traditional method of synthetic fertilizer use is followed worldwide [3]. Surface runoff and leaching are significant factors that lower the nutrient concentrations in soil, and these factors encourage farmers to continuously use chemical fertilizers. This practice may enrich the soils with nutrients; however, the low plant uptake efficiency might be due to a short bioavailability. Additionally, the constant use of chemical fertilizers can negatively affect the soil microbial fauna and flora [4]. Alternatively, the appropriate land use measurements have been extended to fulfil the unique growing requirements to some extent [5]. However, there is still a requirement to comprehend the sustainability at very high environmental costs, thus representing agricultural intensification for production [1]. Biochar has captured the attention of researchers because of its ability to improve the soil cation exchange capacity, the physical and chemical properties of the soil, and the plant nutrient availability [6].

In Australia, biochar and its application to soil have been evaluated for its agricultural and environmental benefits [7]. It has been reported that biochar directly affects the soil fertility, crop production, C sequestration, and fertiliser use efficiency in the soil [8],[9]. The soil fauna and flora, mainly mycorrhizal root colonisation, crop yield, and fertilizer use efficiency, have been reported to improve with biochar incorporation [10]. Additionally, it has been documented that biochar positively impacts arbuscular mycorrhizal (AM) root colonization, crop water use, and nutrient (nitrogen (N) and phosphorus (P)) supply for wheat crops in dryland areas of Western Australia [11]. Many researchers [12]–[14] have also reported positive plant growth and yield response to biochar amendment. The effect of biochar on the soil's physical and chemical properties mainly depends on the pyrolysis process and the feedstock used (e.g., crop residues, animal manures, or wood) [15],[16].

Biochar, a carbon-rich material, is produced by various thermo-chemical biomass conversion processes (i.e., pyrolysis, a dry carbonization procedure that generally takes place at 400–800 °C) [17]. Many studies have shown the variance of potential affirmative consequences of biochar application to soils with some organic fertilizers and beneficial soil microorganisms on the soil properties, crop productivity, and nutrient availability [10],[18],[19]. Biochar positively influences the plant nutrient uptake directly by its mineral composition and release and indirectly by providing better nutrient adsorption [20]. It is also involved in improving the nutrient and water-holding capacity of soils [21],[22], increasing the pH of soils [23], amplifying the physical properties of soil [24], and improving the functions of soil microbial flora and their population in soil [25]. Moreover, the soil amendment with biochar can efficiently use chemical fertilizers by retaining nutrients and by reducing nutrient leaching from the root zone [20]. Biochar has also been used as an excellent option for managing soils that have exhausted their nutrient status, which is now rising as an augmented global concern. As biochar is produced from plant materials, it contains various concentrations of carbon and plant-available macro- and micronutrients. Additionally, the physical and chemical behavior of the biochar produced has a dynamic impact on the plant nutrient uptake capacity by mineralising the nutrients by the soil solution, microbial biomass, and plant roots [26],[27]. For the current experiment, the biochar was produced from wheat straw and was characterised, and its impact on the soil moisture availability to the crop, carbon sequestration, crop growth, and yield in rainfed conditions were studied.

Phosphorus plays essential roles in AM colonization and rhizobium nodulation processes. Additionally, biochar's positive roles in AM and nodulation are articulated. However, the effects of the co-application of biochar and P on AM colonization and rhizobium nodulation were poorly studied.

## 2. Materials and methods

The field soil used in this experiment was an acidic white Tenosol [28] and ‘Dystric Fluventic Eutrudept’ as reported in the US Soil Taxonomic classification [29] at Moonyoonooka near Geraldton, Western Australia (28°44′11.94″S 114°43′15.84″E). The experimental location has a Mediterranean-type climate with a mean annual rainfall and temperature of <400 mm and 28 °C, respectively. Within the year of this trial, the experimental site received about 245 mm of rainfall. The soil used for the experiment was preferred because of its loamy sand texture, which provides a low N and P retention capacity. This is the representative property of most soils in the Western Australian agricultural area.

In this experiment, wheat straw biochar in three rates (0, 5, and 10 t ha^−1^ noted as B0, B5, and B10, respectively) was used without and with the application of different levels of P fertilizer (0, 5, and 10 kg P ha^−1^ noted as P0, P5, and P10, respectively) [10],[11], while all the polyvinyl chloride (PVC) tubes filled with soil (collected from 0–10 cm) were inoculated with rhizobium inoculum. The soil was mixed with biochar and P by weight and left undisturbed for one month before subterranean clover seeds were sowed. Polyvinyl chloride tubes, each with a dimension of 1 m long and a diameter of 100 mm with both ends opened, were used in a randomized block design under open-air conditions. Subterranean clover (*Trifolium subterraneum* cv Dalkeith) seeds at the rate of ten seeds per tube with applied treatments were sown but were thinned to four seedlings after two weeks of germination. Dalkeith is an early flowering subterranean clover best suited to moderately acidic, well-drained soils with an annual rainfall of approximately 300–500 mm. The growing period was from early October to December.

### 2.1. Soil and plant measurements

The 0–10 cm soil layer was analyzed for basic properties (Table 1). The soil contained 85% sand, 3% silt and 12% clay. The pH of the soil was 4.8 and was measured at 0.01 M CaCl_2_ at 1:5 (w/v) ratio. The organic matter was 10.3 g kg^−1^ in the soil measured by dry combustion using an elementar (vario MACRO CNS; Elementar, Germany). The soil contained 0.6 g kg^−1^ total N, and 7 and 5 mg kg^−1^ NO_3_-N and NH_4_-N, respectively. This soil was chosen because of its loamy sand texture, which gave it a low capacity to retain P, and the low available P (7.5 mg kg^−1^) content was suitable for a mycorrhizal response in the subterranean clover. The soil P and K were measured using the 0.5 M NaHCO_3_ extraction method [30]. The available soil Colwell P, organic P [31], and microbial biomass P [32] were extracted after harvesting the plants. The P concentration in the extracts was measured using a spectrophotometer [33]. After 111 days of growth from each treatment pot, the sown crop was harvested and separately placed into a plastic bag as shoots and roots. Then, the soil and plant samples were brought into the laboratory and oven-dried at 70 ºC for 72 h for analysis. A sub-sample of the fresh roots was fixed in a formaldehyde mixture (ethanol 70%, formaldehyde, and acetic acid at a ratio of 90:5:5 v/v) to count the nodulation. The oven-dried shoots were ground and digested in a 3:1 HNO_3_-HClO_4_ mixture, and the P concentration within the digest was measured by the molybdenum-blue method. The P shoot uptake was calculated by multiplying the shoot P concentration by the shoot weight.

**Table 1.** Basic properties of wheat straw biochar and soil used in this experiment.

Properties	Wheat straw biochar	Soil
Pyrolysis temperature (°C)	450	-
pH (H_2_O)	8.4	5.8
pH (CaCl_2_)	8.3	4.9
EC (dS m^−1^)	9.2	0.05
Carbon (g kg^−1^)	531.0	13.1
Nitrogen (g kg^−1^)	2.2	1.0
C:N ratio	241.4	13.2
NH_4_^+^-N (mg kg^−1^)	2.1	5.0
NO_3_^-^-N (mg kg^−1^)	<0.1	7.0
Phosphorus (mg kg^−1^)	0.4	7.5
Potassium (mg kg^−1^)	3.4	35
CEC (m.e./100gC)	45.6	4.0
WHC (%)	287	25

### 2.2. Assessment of mycorrhizal colonization

The root colonization by the AM fungi was estimated using the standard procedure described by [34]. Briefly, the root samples (0.5 g fresh weight) were cleaned by treating them with a 10% KOH solution, rinsed with water, acidified with 10% HCl, and then stained with 0.05% trypan blue. The root colonization in the subterranean clover plants was determined by examining the percentage of the root length colonized under a dissecting microscope using the gridline intersects method [35]. The gridline intersects were counted for the presence of mycorrhizal structures at ×100 magnification. Mycorrhizal colonization was counted in three replicate samples.

### 2.3. Statistical analyses

Genstat (v.18) was used to conduct all the statistical analyses. A two-way analysis of variance (ANOVA) was used to determine the significant difference among the applied treatments, and Fisher's protested least significant difference (LSD) was applied to check for any significance between the means of the results. The significant Pearson correlations between the measured soil and plant parameters were tested after eight weeks of subterranean clover growth. Additionally, the plant parameters (shoot and root mass, P concentration, and uptake in shoots) and mycorrhizal colonization were explored with a principal component analysis (PCA) to identify the association between these traits and mycorrhizal colonization. The analysis was based on the correlation matrix and multivariate analyses performed for the plant parameters with the mycorrhizal root colonization [36].

## 3. Results

### 3.1. Effect biochar and P on growth, root nodulation, and colonization of subterranean clover

**Figure 1.**
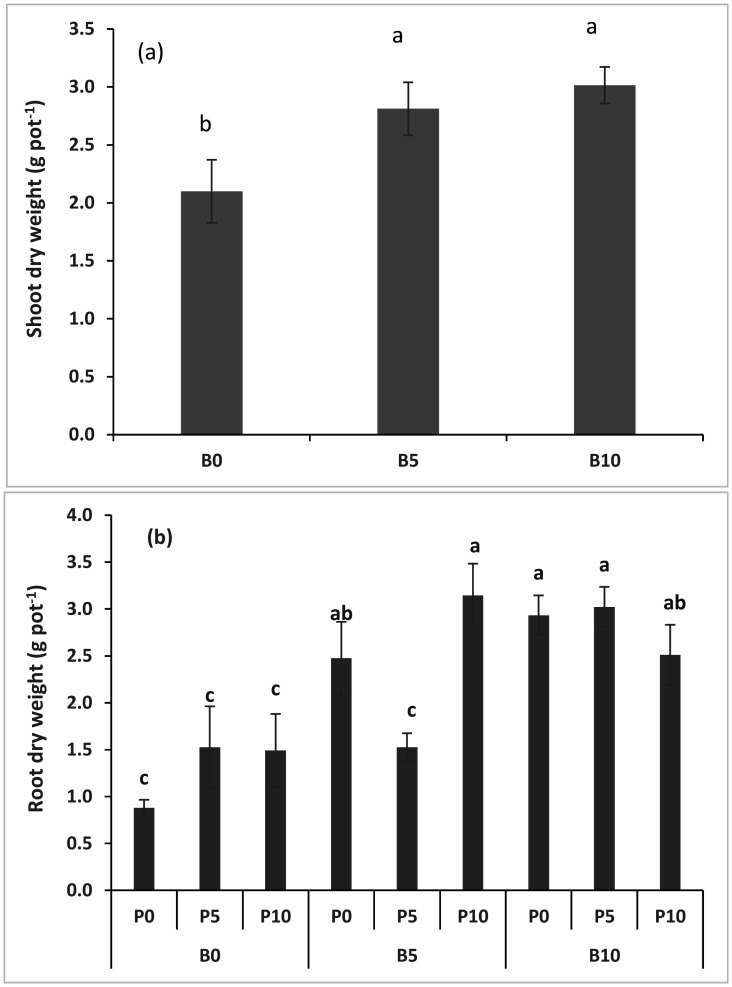
Effect of wheat straw biochar rates on (a) the shoot dry weight, and wheat straw biochar × P fertilizer interaction effect on (b) the root dry weight of subterranean clover in the field conditions.

Data of the shoot and root dry weight of subterranean clover are presented in Figure 1. The interaction effect of biochar and the P fertilizer on the shoot dry weight was not significant. The main effect of biochar on the shoot dry weight was significant when the B5 and B10 were higher than the control (Figure 1a). Biochar and the P fertilizer interactions significantly affected the root dry weight. It showed a maximum root dry weight in treatment B5P10, followed by B10P5, which was statistically similar to B5P0, B10P0, and B10P10. In contrast, the lowest root dry weight was obtained in the treatment where no fertilizer and biochar were applied (Figure 1b). The main effect and the interaction effect of biochar on the shoot-root ratio were insignificant (data not shown).

**Table 2.** Results of *p* values of two-way ANOVA showing the significance level of all treatment combinations (with four replications).

Sources of variations	Shoot DW	Root DW	S/R ratio	Myc. Colon.	Nodulation	Shoot P conc.	Shoot P uptake	Soil available P	Microbial biomass P	Soil organic P
Biochar	**<0.001**	<0.001	0.962*ns*	<0.001	**0.014**	<0.001	<0.001	0.141*ns*	0.129*ns*	**0.043**
P	0.177*ns*	0.369*ns*	0.600*ns*	<0.001	0.255*ns*	0.258*ns*	<0.001	**0.014**	<0.001	0.640*ns*
Biochar × P	0.087*ns*	**0.010**	0.386*ns*	**<0.001**	0.475*ns*	**0.042**	**<0.047**	0.549*ns*	**0.026**	0.628*ns*

*Myc. = mycorrhizal; Colon. = colonisation; conc. = concentration; ns = not significant*

**Figure 2.**
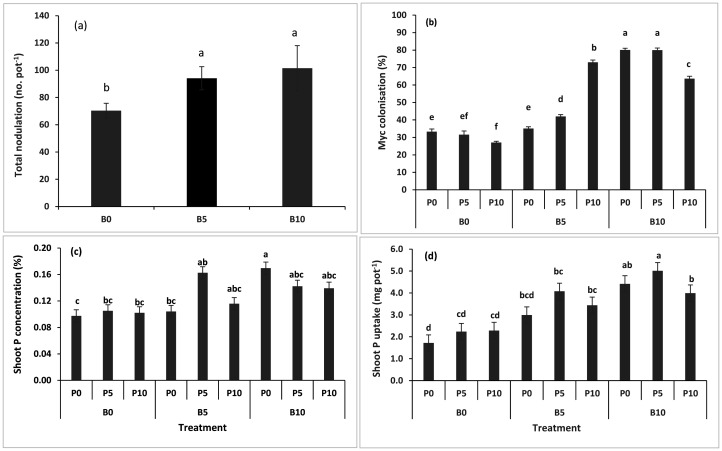
Effect of wheat straw biochar on (a) the total nodule number and biochar × P fertilizer interactions effects on the (b) root colonisation, (c) shoot phosphorus concentration, and (d) shoot phosphorus uptake of subterranean clover in the field conditions.

The result of the total nodule number per pot of the subterranean clover shows that the nodule number significantly increased with the addition of biochar, irrespective of the biochar rates to the control (Figure 2a). The mycorrhizal root colonization also showed similar trends. The data shows that biochar integration improved the arbuscular mycorrhizal fungi associations with the crop roots (Figure 2b). The mycorrhizal root colonization was significantly (p ≤ 0.005) higher in response to the separate fertilizer and biochar applications, and even shows a higher significance level (p ≤ 0.001) in a combination of both biochar and the P fertilizer (Table 2).

### 3.2. Effect on plant P concentration and uptake

Data regarding the clover plants' P concentration and its uptake by plant shoots increased when biochar was added to the plant. The result indicates that the highest P concentration was noted in treatments where B10P0 and B5P5 were applied, which were not significantly higher than the B5P10, B10P5, and B10P10 treatments. All these treatments showed statistically similar percentiles of P in the clover shoot. Still, without the addition of biochar, the fertilizer application did not increase the shoot P percentile (Figure 2c). A similar trend was noted in the case of P uptake of the clover shoots, where treatments B10P5, B10P0, B5P5, B10P10, and B5P10 showed an increase of P uptake by the shoot as compared to the other applied treatments (Figure 2d). The biochar application significantly increased the P concentration in the shoots, as well as the P uptake by the shoots. Still, applying the fertilizer did not affect the P concentration in shoots nor its uptake by the plants.

### 3.3. Effect on available, microbial and organic P concentration in soil

**Figure 3.**
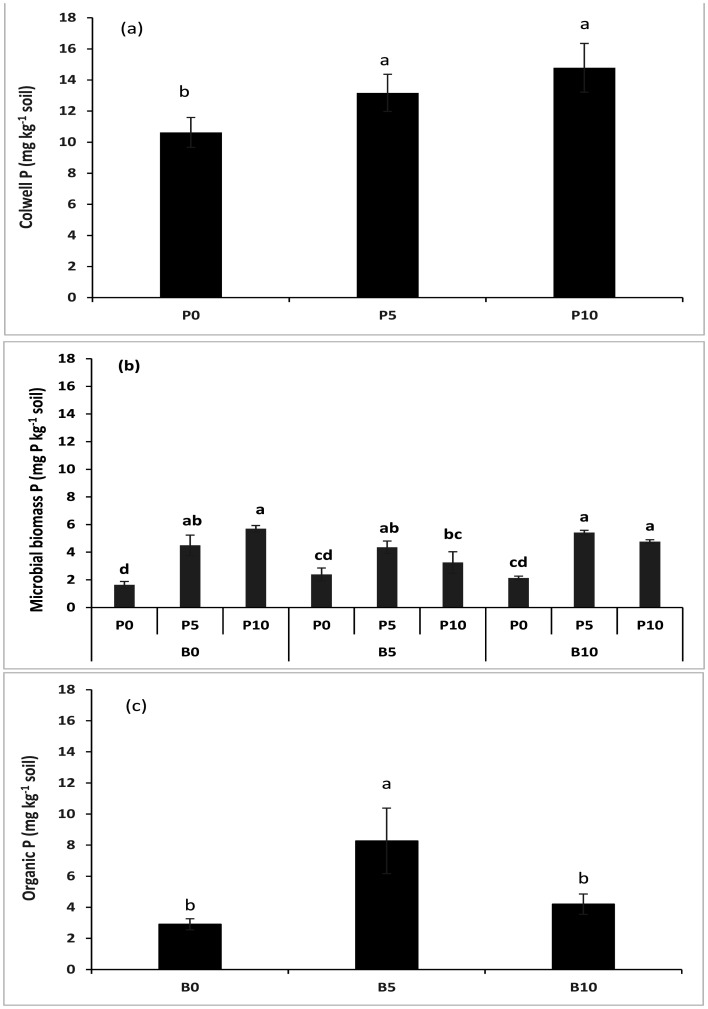
Effect of P fertilizer on soil (a) the available phosphorus, biochar × P fertilizer interactions effect on (b) the microbial biomass phosphorus, and biochar effect on (c) the organic phosphorus concentrations after harvest of subterranean clover in field conditions.

Data related to the available microbial and organic P is represented in Figure 3. At the harvest of the subterranean clover, it was observed that the Colwell available P was noted to be maximum in the B10P5, B10P10, B5P10, and B0P10 treatments as compared to the other fertilizer treatments; this increase was statistically similar in all treatments. In contrast, the lowest was noted in pots where no biochar or fertilizer was applied (Figure 3a). A similar trend was observed in the microbial P in the soil of the clover rhizosphere area. It was noted that the application of the B0P10, B5P5, B10P5, and B10P10 treatments showed increased microbial P concentrations compared to the other treatments where a low dose of the fertilizer was used (Figure 3b). The biochar application with the combination of the fertilizer application probably increases the microbial activity. As a result, it significantly increased the available and microbial P. On the other hand, the organic P in the soil showed an increase only in the B5 treatment (Figure 3c), and no significant interactions were observed between the biochar and P treatments applied.

### 3.4. Correlations between soil or biochar properties and plant parameters

Significant correlation coefficients (r) were observed between the soil P fractions, all the measured plant growth parameters, and the % mycorrhizal colonization (Table 3). The soil available P (NaHCO_3_-extractable Colwell P) was significantly correlated with the nodulation numbers (r = 0.426*, p ≤ 0.05), the shoot P uptake (r = 0.444*, p ≤ 0.05), and the mycorrhizal root colonization (r = 0.309*, p ≤ 0.05) in the subterranean clover plants (Table 3). A significant correlation coefficient (r) was also observed between the nodulation number with the shoot DW (r = 0.457*, p ≤ 0.05) and with the uptake of P in the plant shoot tissues (r = 0.528*, p ≤ 0.05). Even though the availability of P in the soil and the % mycorrhizal colonization were positively correlated, the soil microbial biomass and the soil organic P were not significantly correlated with the % mycorrhizal colonization, plant growth, or P uptake (p ≤ 0.05). The shoot dry weight, shoot P concentration, and shoot P uptake were positively correlated with the % mycorrhizal colonization for the subterranean clover (p ≤ 0.05). There was a non-significant correlation between the root biomass of the subterranean clover and mycorrhizal colonization (p ≥ 0.05) and between the root biomass with a nodulation of the subterranean clover (p ≥ 0.05). However, there was a non-significant positive correlation between the nodulation and the % mycorrhizal colonization of the subterranean clover (r = 0.232, p > 0.05).

**Table 3.** Pearson's correlation coefficient (r) between the mycorrhizal colonization, nodulation, soil, and plant properties after 16 weeks of growth.

Variables	Shoot DW	Root DW	Nodulation number	S/R ratio	Soil available P	Microbial biomass P	Soil organic P	Shoot P conc.	Shoot P uptake
Shoot DW	1								
Root DW	**0.713**	1							
Nodulation	**0.457**	0.295	1						
S/R ratio	-0.097	**-0.630**	-0.115	1					
Soil available P	0.136	0.046	**0.426**	0.070	1				
Microbial biomass P	-0.015	0.174	0.153	-0.291	0.136	1			
Soil organic P	-0.050	-0.081	0.137	0.056	0.038	-0.144	1		
Shoot P conc.	-0.222	**-0.317**	0.097	**0.322**	0.276	0.015	-0.172	1	
Shoot P uptake	**0.609**	**0.350**	**0.528**	0.127	**0.444**	0.146	-0.230	**0.560**	1
Mycorrhizal col.	**0.375**	0.193	0.232	0.174	**0.309**	-0.048	-0.255	**0.563**	**0.690**

Values in bold are significant at level *p* = 0.05. conc. = concentration

### 3.5. Principal component analysis (PCA) revealed patterns in plant parameters across all treatments

**Figure 4.**
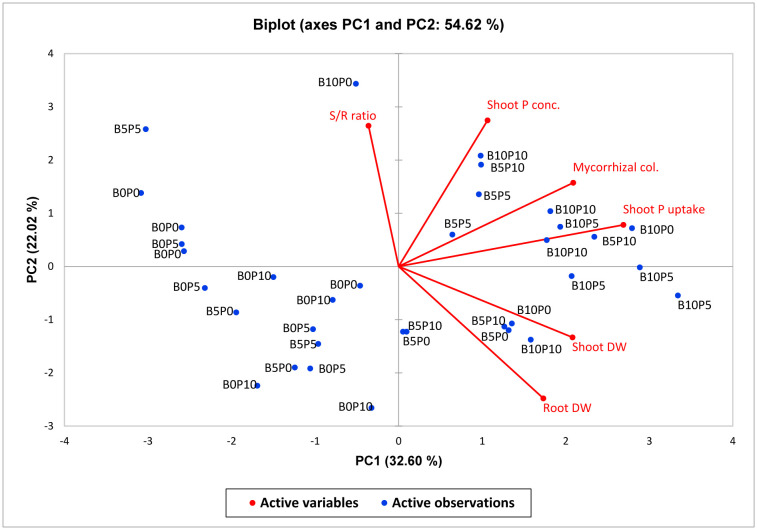
Biplot of principal component analysis ordination diagram based on PC1 vs PC2 of the subterranean clover (mycorrhizal colonization, shoot and root mass, S/R ratio, P concentration and P uptake in the shoots) for samples of the biochar and P fertilizer treatments plus a control. The percentage of the total variance, as explained by each axis, is shown.

The principal component analysis biplot in Figure 4 showed a correlation among different parameters of the study observed. They include P concentrations in the shoots and roots of the subterranean clover, mycorrhizal colonization, shoot and root dry mass of plants, and their ratio. Variables close enough and forming a slight angle are positively correlated, and those far away from each other at an angle of 180° are negatively correlated. With the application of all treatments, PC1 and PC2 were most influenced by all the treatments compared to control, which positively influenced the plant characteristics and P concentration in the shoots and roots. The scatterplot showed slightly overlapping groups of samples corresponding to the particular treatments applied, and the differentiation pattern concerning the plant parameters was pronounced with the treatments used. The most significant differences between biochar and the P fertilizer rates were observed for the subterranean clover. The observed increased % of mycorrhizal colonization in the subterranean clover corresponded to an enhanced P concentration with both the shoots and roots.

## 4. Discussion

### 4.1. Effect on growth, root nodulation and colonization of subterranean clover

Data of the shoot and root dry weights of the subterranean clover and their ratio revealed a significant increase with the biochar application; additionally, a significant improvement could be observed in the cases of the total dry weight, mycorrhizal colonization, and nodule numbers. Adding biochar with the fertilizer and microbial inoculation was reported to increase the soil's crop growth and nutrient uptake capacity [10],[37],[38]. The mechanism behind this increase is the availability of inorganic and organic nutrients present in biochar and those from inorganic fertilizers. A significant part of N also contributes to the developed nodulation system of roots. Moreover, many other studies revealed that the application of biochar induced the expression of specific genes that could influence plant growth [39],[40]. The dual inoculation of *Rhizobium* and *Glomus* has also been reported to significantly increase the soybean plants' total biomass [40]. Our findings indicated that the subterranean clover had produced more mycorrhiza colonized roots in the pots when low amounts of biochar and fertilizer were applied. The use of biochar reduced the amount of fertilizer required and increased the root nodulation and AM fungal root colonization. A detailed study on biochar clearly stated that the soils amended with biochar could modify the soils' physicochemical properties, which may lead to increases in the soil pH and nutrient availability and subsequent alterations in the root colonization by AM fungi [41].

In their studies, many researchers quoted the positive impact of biochar on root colonization and documented that the P supply can be enhanced by the addition of biochar in the drylands of Western Australia. Biochar has the capacity to build up the mycorrhizal colonization in soil and, in return, influence the P and N uptake of the plants. The amendment of soil with biochar and microbial inoculation was reported to increase the mycorrhizal colonization of crop roots and their growth in rainfed areas of Western Australia [10],[11].

### 4.2. Effect on P concentration and uptake of subterranean clover

Data regarding the P concentration and its uptake by the subterranean clover plants showed an increase with biochar and the P fertilizer application. The increase in the plant P concentration and uptake may be possibly due to the application of biochar with the P fertilizer and the stimulation of the mycorrhizal colonization, thus leading to an increased P uptake. Phosphorus uptake by plants largely depends on the beneficial associations of arbuscular mycorrhizal fungi with the roots of plants. These fungi release some extracellular phosphatases and P-solubilizing organic acids, thus making the organic P plant available. Many scientists have reported that adding biochar boosts the mycorrhizal colonization of roots by producing favorable environmental conditions for mycorrhizal spores' survival. Thus, the plant P uptake occurred with an enhanced solubility of P [41]–[43]. The addition of biochar to soils improves the N, P, and other major cation concentrations in soils, as well as their availability and uptake by plants [10],[44]. The response of biochar is associated with the types and properties of the biomass used in the production of biochar, the addition of chemical and organic fertilizers to the soil, and the crops grown [6],[20],[45]. The addition of biochar to the rhizosphere of the P-deficient soils has the potential to increase the plant growth, yield, and P uptake [46].

### 4.3. Effect on soil available, microbial and organic P concentration

Data on the post-harvest soil available and microbial biomass P concentrations showed a significant increase with the P application but not with biochar. This slow release of P could be due to the application of biochar, which may capture the available P on its binding sites and slowly release it to the crop. The same level of soil P concentration in higher biochar rates may be due to the low amount of available P in biochar. Many previous studies have revealed that P and N may become either more available or unavailable to plants and soils after the soil amendment with biochar, which largely depends on the nutrient concentrations of the applied biochar [7],[41],[47]. The available organic and microbial P concentrations increased with the addition of biochar and the P fertilizer. The increase in soil P concentration could result from the application of biochar because biochar can change the soil's physicochemical properties, thus leading to an increased nutrient availability in soil [41],[44],[48].

## 5. Conclusions

Based on the results, adding biochar to soils with a P fertilizer increased the biomass of subterranean clover roots, whereas biochar increased the shoot dry weight more than the control irrespective of the P fertilizer rates. In addition, the increased root colonization induced more P uptake of plants when biochar with a mineral P fertilizer was applied. The research results also indicated the positive roles of biochar and the P fertilizer to provide more economical access to P as either applied or stored in the soil and to help improve the profitable clover pasture production. It is concluded that the combined application of biochar and a mineral P fertilizer can increase the microbial activities and decrease the use of chemical fertilizers without compromising the environmental risks and improving the sustainability of agriculture. However, more research under field conditions is necessary for a quantitative recommendation.
